# Analysis of the Influence of Crystalline Admixtures at Early Age Performance of Cement-Based Mortar by Electrical Resistance Monitoring

**DOI:** 10.3390/ma14195705

**Published:** 2021-09-30

**Authors:** Rubén Beltrán Cobos, Fabiano Tavares Pinto, Mercedes Sánchez Moreno

**Affiliations:** Departament of Química Inorgánica, Instituto de Investigación en Química Finay Nanoquímica, Universidad de Córdoba, Campus de Rabanales, 14071 Córdoba, Spain; rbeltran@uco.es (R.B.C.); ftavares@uco.es (F.T.P.)

**Keywords:** crystalline admixtures, initial setting time, early age performance, wireless electrical resistance, waterproofed concrete

## Abstract

Crystalline admixtures are employed for waterproofing concrete. This type of admixtures can affect the early age performance of cement-based mixes. The electrical resistance properties of cement have been related to the initial setting time and to the hydration development. This paper proposes a system for remote monitoring of the initial setting time and the first days of the hardening of cement-based mortars to evaluate the effect of the incorporation of crystalline admixtures. The electrical resistance results have been confirmed by other characterization techniques such as thermogravimetric analysis and compressive strength measurements. From the electrical resistance monitoring it has been observed that the incorporation of crystalline admixtures causes a delay in the initial setting time and hydration processes. The measurements also allow to evaluate the influence of the amount of admixture used; thus, being very useful as a tool to define the optimum admixture dosage to be used.

## 1. Introduction

Crystalline admixtures (CAs) are proposed as an effective alternative for waterproofing concrete when watertight conditions are needed [1]. Their action mechanism is based on the ability for reacting with the water and cement particles, increasing the density of the solid matrix in concrete [2,3] and hindering the penetration of external water into the structure [4]. The full waterproofing effect of concrete prepared with a crystalline admixture has been reported to occur several days after casting [5].

Different aspects on the waterproofing performance of concrete incorporating crystalline admixtures have been evaluated by water absorption tests [6], chloride penetration [7] and permeability tests [2,3]. It has been reported that the efficiency of these compounds in preventing water penetration into the concrete is related to their ability react with portlandite (CH) promoting the formation of non-soluble compounds and sealing the pores inside the concrete bulk [8]. Recently, Oliveira et al. [9] studied the performance of crystalline admixtures during the early age when added to cement pastes. They reported a retard on the hydration processes when CA added to cementitious mixes, more significant with the increment in CA dosage. However, no studies have been found in the literature to evaluate the effect that this delay in the hydration processes may have on the properties of the fresh state of the mix or on the mechanical strength of the concrete.

In fact, the fresh state of a cement-based mix determines its further performance at the hardened state [10]. Thus, being able to know how the presence of the crystalline additive affects the behavior of the mixture in the first curing ages can be very useful when dosing the amount of additive to be used. The reference method for determining the setting time of cement is the needle penetration test [11]. However, other non-destructive methods have been also proposed for the cement setting assessment, such as the ultrasonic wave transmission method [12,13,14,15] or acoustic emission methods [16,17,18].

The electrical resistivity of cement has been related to the setting time of cement-based mixes [19,20,21]. The chemical and physical changes occurring during cement hydration affect the resistivity development [22,23]. Yousuf et al. [24] proposes monitoring electrical resistivity of cement pastes for evaluating the setting and hardening behavior of cement pastes incorporating different types of additives using a non-contact method for the resistivity measurements, with continuous automatic data recording using a computer. The authors concluded the suitability of electrical resistivity as a method for the visualization of microstructure development in cement pastes at early ages. Electrical resistivity has also been used to evaluate the influence of different admixtures on the fresh state behavior of cement-based mixes [25,26,27].

In the present study the continuous monitoring of the electrical resistance of mortars containing different amounts of crystalline admixtures is proposed as method for evaluating the effect of the additives on the setting and hardening processes. An integrated system for the remote monitoring of the electrical resistance was employed.

The proposed system can be used as a tool for assessing the quality of cementitious mixes from an early age as well as for optimizing the dosage of additives that, when incorporated in the mix, may affect its behavior in the fresh state.

## 2. Materials and Methods

### 2.1. Specimen Preparation

Prismatic mortar samples, 40 × 40 × 160 mm^3^, with embedded sensors for monitoring the electrical resistance response were prepared. Two different cases were considered: a reference mortar without any admixture and a waterproofed mortar incorporating different contents of a commercial hydrophilic crystalline admixture. Three sensors were considered for each case to evaluate the repeatability of the measurements.

Ordinary Portland cement (OPC) CEM II/S-V 42.5R, normalized siliceous sand (0–4 mm) and tap water were used for the sample preparation. A fixed w/c ratio of 0.5 was maintained for all the cases. Two different dosages of the crystalline admixture (CA) were added to the mortar, as recommended by the supplier (0.9% by cement weight, ADD-1) and below the recommended dosage (0.45% by cement weight, ADD-0.5) to evaluate the influence of the additive content in the cementitious mix. The mortar mix proportion is detailed in Table 1.

The mortar mixes were prepared according to the Spanish standard EN 196-1:2005 [28]. Polymeric molds were used for avoiding interference in the electric resistance measurements of the sensors. The samples for mechanical testing were casted in metal forms.

The mortar samples were curing under controlled conditions (22 ± 2 °C, >98% RH). Two different curing ages were considered for characterizing the mortar properties, 2 and 7 days. At these curing ages, the behavior of the mortars was characterized. On the one hand, the compressive strength was measured and on the other hand, the hydration of the solid phases was analyzed. For this purpose, the hydration of the cement was stopped at the desired ages by replacing the pore water with ethanol and subsequent vacuum drying with acetone [29]. The samples were kept in a desiccator until the analysis was carried out. Finally, after 7 days of curing, the waterproofing capacity of the mortars was also evaluated by the capillary water absorption test.

### 2.2. Monitoring of Electrical Resistance during Setting and Curing

**Figure 1:**
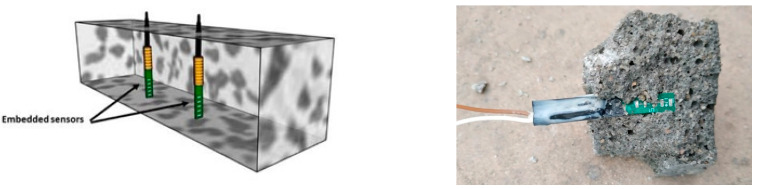
Scheme (**left**) and photography (**right**) of the sensors embedded in the mortar.

Sensors for monitoring the evolution of the electrical resistance in the bulk of the mortar samples were specifically designed and fabricated for the study. In Figure 1, a small print circuit board (PCB) with five tin-plated metal points of the same size (2.4 mm^2^), separated a constant distance of 2.5 mm, was used. The two opposite points closest to the end of the PCB were used for measuring. The design of the sensor is based on the idea of further application to assess the water penetration at different depths in the mortar bulk.

The prototype was based on a development board Teensy 3.6 to read the resistivity sensor once a minute. The sensor values were registered into a native micro-SD card port. To guarantee the proper work of the equipment, a Bluetooth module was installed to send the data in real time to a mobile device as a serial terminal. For the need to read several sensors with the equipment in continuous mode a 32-nodes home-fabricated multiplexer module was developed. It was used 8 ADG511 as monolithic CMOS ICs containing four independently selectable analogical switches each, driven by four 74HC595 as high-speed CMOS 8-bit shift register.

The measurements were carried out determining the charge/discharge time of a capacitor with known capacitance when an AC voltage is applied. The charge/discharge frequency is directly related to the electrical resistance of the mortar through a previous calibration procedure. The electrical resistance was monitored from the manufacturer of the specimens for up to 7 days of curing.

### 2.3. Characterization of the Specimens

#### 2.3.1. Initial Setting Time

A VICAT manual apparatus was used for determining the initial setting time of the studied samples as this is the standard test for measuring this parameter in cement paste and mortar [30]. During the needle penetration tests the specimens were maintained in the camera at high-humidity conditions.

#### 2.3.2. Compressive Strength

The specimens were broken in two halves by a three-point bending test. The compressive strength was tested on each of the halves obtained, so that four measurements were considered for each type of mortar tested. The mean value is shown in the results of the present study.

#### 2.3.3. Hydration Development

As mentioned above, at 2 and 7 days of curing, the development of the cement hydration processes in the different mortars studied was analyzed. Thermogravimetry tests (TG/DTG) were used for this purpose. The equipment used for the thermogravimetric analyses was a TGA/DSC 1 Star System Mettler Toledo thermobalance with a heating rate of 10 °C/min, between 30 and 1000 °C. The atmosphere was oxygen with a flow rate of 100 mL/min. The sample holders were 100 μL aluminum crucibles.

#### 2.3.4. Waterproofing Efficiency of the Crystalline Additive

The waterproofing ability of the crystalline additive was assessed after 7 days of curing under high-humidity conditions. Measurements were carried out according to the Fagerlund method [31]. The weight gain of the specimen in contact with a 5 mm high sheet of water was measured periodically, and the saturation time (t_n_) and the water capillary absorption coefficient (k) were determined using the standard procedure [31].

For each type of mortar tested, the average value of three specimens was considered.

## 3. Results and Discussion

### 3.1. Evolution of Electrical Resistance during the 1st Day Curing

#### 3.1.1. Reference Case

**Figure 2:**
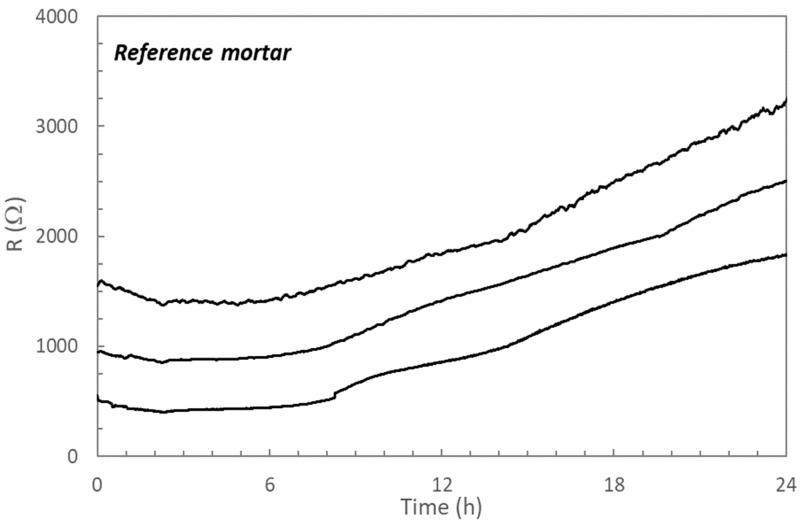
Evolution of the electrical resistance during the first curing day of the reference mortar for the three different sensors embedded in the specimen.

The evolution of electrical resistance values of three different sensors embedded in the reference mortar is shown in Figure 2.

Although the absolute values of the different sensors vary, the evolution of the electrical resistance with the curing time is similar. It can be observed that during the first hours of hydration the values of the electrical resistance slightly decrease until a minimum is reached at about 2.5 h. The dissolution of species from the unhydrated cement particles can be expected during this first period of hydration, explaining the decrease in the resistance values [32]. Once the setting starts, the formation of the initial nucleus of solid phases occurs [33], counteracting the effect of the dissolution of the species, changing the tendency of the resistance evolution which maintain in almost constant values. These two opposite behaviors explain the almost constant value maintained during several hours. After about 8–10 h of hydration, a steady increase in the electrical resistance with time was registered in all sensors. This evolution must be indicating the continuous formation of the hydrated phases of the cement paste [34].

From Figure 2 the sensitivity of the embedded sensors to the hydration evolution of the cement paste can be deduced, even though if the absolute values of the sensors differ, starting between 500 and 1500 Ω and finishing, after 1 day of hardening, between 1500 and 3000 Ω depending on the sensor.

#### 3.1.2. Waterproofed Mortar Containing Crystalline Admixtures

**Figure 3:**
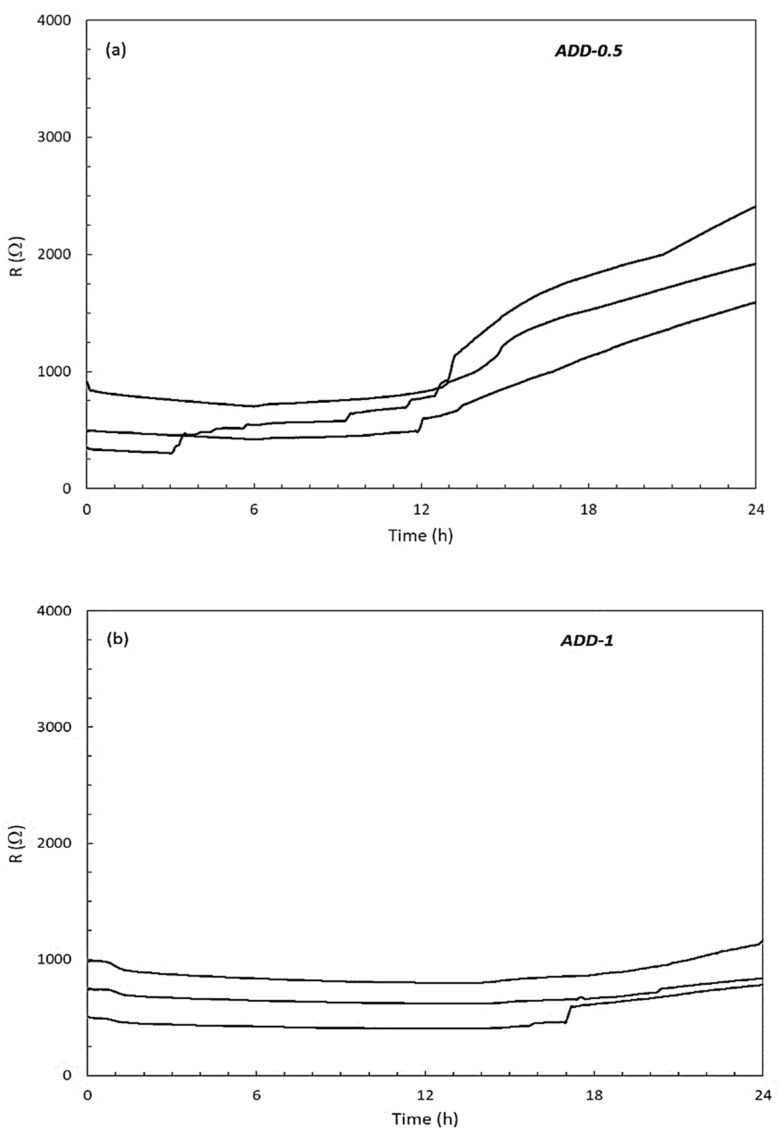
Evolution of the electrical resistance (three sensors per case) during the first curing day of mortar with different content of crystalline admixture. (**a**) 0.45%, (**b**) 0.9%.

In the mortar incorporating the crystalline admixture, a similar evolution was observed during the first day of the cement paste hydration, as can be seen in Figure 3a,b, related to a mortar with an additive content of 0.45% (ADD-0.5) and 0.9% (ADD-1), respectively. In the presence of the crystalline additive, significantly lower values of electrical resistance are recorded than in the case of the reference mortar during the first day of hydration.

Similar to what happened in the case of the reference mortar during the first hours of hydration, a slight decrease in the electrical resistance values was registered in both cases, until reaching a minimum. However, in the presence of the crystalline additive the period of constant values of electrical resistance before starting the steady increase was longer than in the reference mortar. Depending on the additive content, a delay of about 12 h of hydration occurs in the presence of 0.45% of additive (ADD-0.5), and in the presence of 0.9% of additive (ADD-1), even after 1 day hydration the increase on the resistance values are still slow.

In Table 2 the mean values of the initial electrical resistance values of the sensors for each case of study, and the mean values after 1 day of curing are summarized. Values in Table 2 reflect that when mortars incorporate the additive significantly lower electrical resistance values are registered from the beginning of the mix; these lower values are maintained even after 1 day of curing. During the first day of hardening, a slower increase in the electrical resistance values is registered for the mortars containing the additive, this effect is more significant when a higher content of additive is added to the mix (ADD-1).

The slower increase in the electrical resistance values when 0.9% of additive (ADD-1) is added to the mix must be related with the action of the additive hindering the formation of the hydrated phases of the mortar sample.

### 3.2. Initial Setting Time: VICAT Measurements

**Figure 4:**
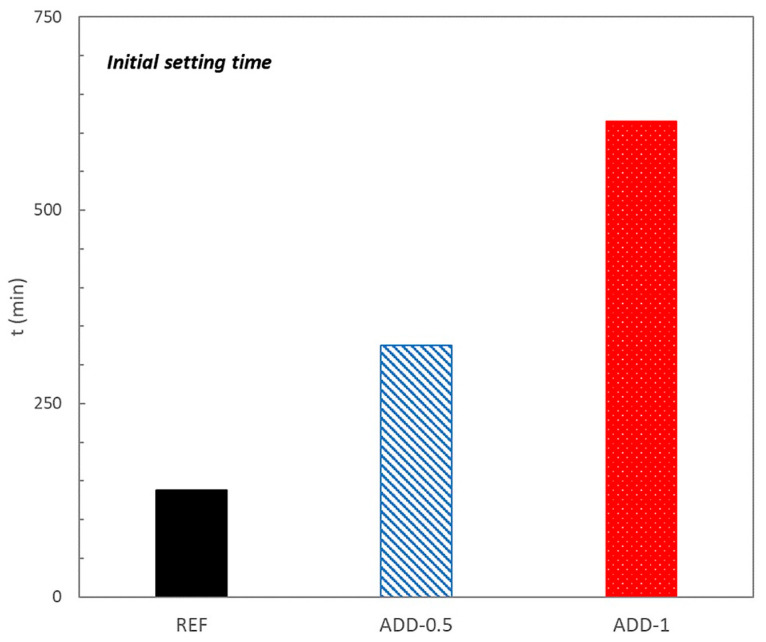
Initial setting time estimated by VICAT needle test.

The effect of the crystalline additives on the initial setting time of the mortars, determined with VICAT apparatus is presented in Figure 4.

When crystalline admixture is present in the mixture a delay in the initial setting time is observed. Higher additive content promotes a longer delay from the beginning of the mix setting.

Data obtained with the VICAT needle test for the reference mortar were compared with the electrical resistance. The normalization method (rescaling real value numerical attributes into the range 0 and 1) was applied on the electrical resistivity to improve the visualization of different sensor positions. The following equation was used:

(1) $$x_{normalized}=\frac{x-x_{min}}{x_{max}-x_{min}}$$

**Figure 5:**
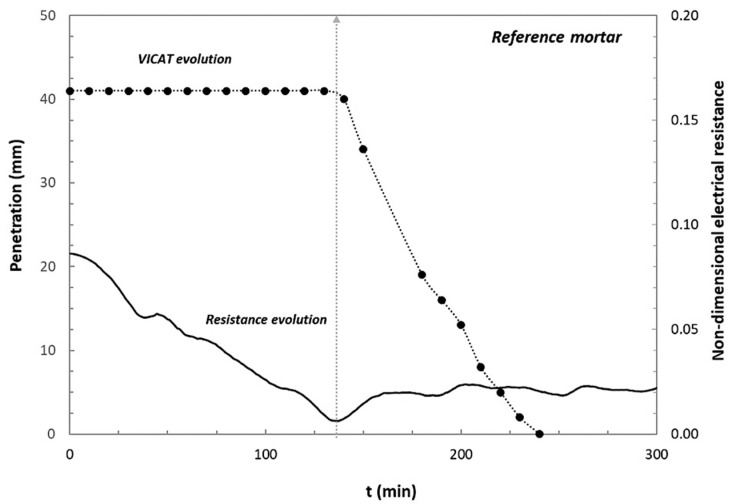
Comparison between needle penetration during VICAT test and electrical resistance evolution for the reference mortar.

The comparison of the needle penetration during VICAT test with the mean values of the non-dimensional electrical resistance values is represented in Figure 5 for the specific case of the reference mortar.

A clear correspondence between the initial setting time and the time at which is reached the minimum value of resistance can be observed, approximately after 120 min from mixing.

**Figure 6:**
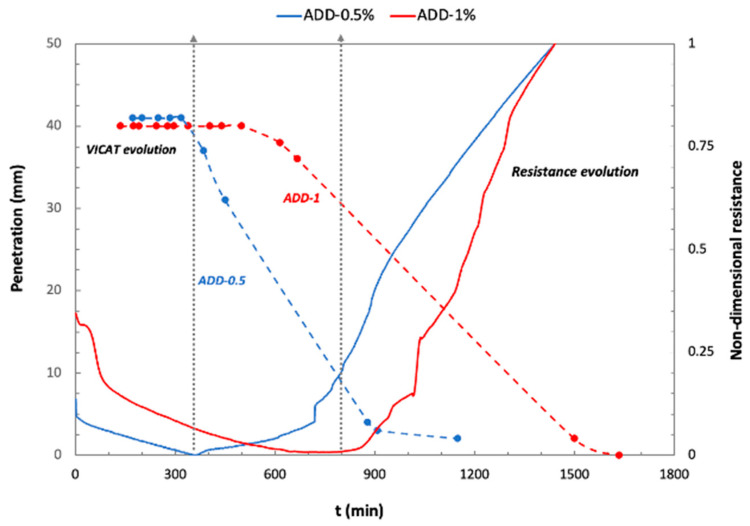
Comparison between needle penetration during VICAT test and electrical resistance evolution for the mortar with crystalline admixtures.

The presence of the crystalline admixture in the cementitious matrix induces a delay in the initial setting time, as was previously mentioned (Figure 4). When comparing the results of the needle penetration and the evolution of the electrical resistance for the mortars with the crystalline additive, as shown in Figure 6 a delay in the minimum reached by the resistance with respect to the initial setting time determined by the VICAT test is observed, with a more prominent effect observed for higher crystalline additive content. A deceleration of the cement clinker dissolution was reported for mortars incorporating a retarder admixture in the mix [35].

### 3.3. Influence of the Crystalline Admixture on the Hydration Processes

**Figure 7:**
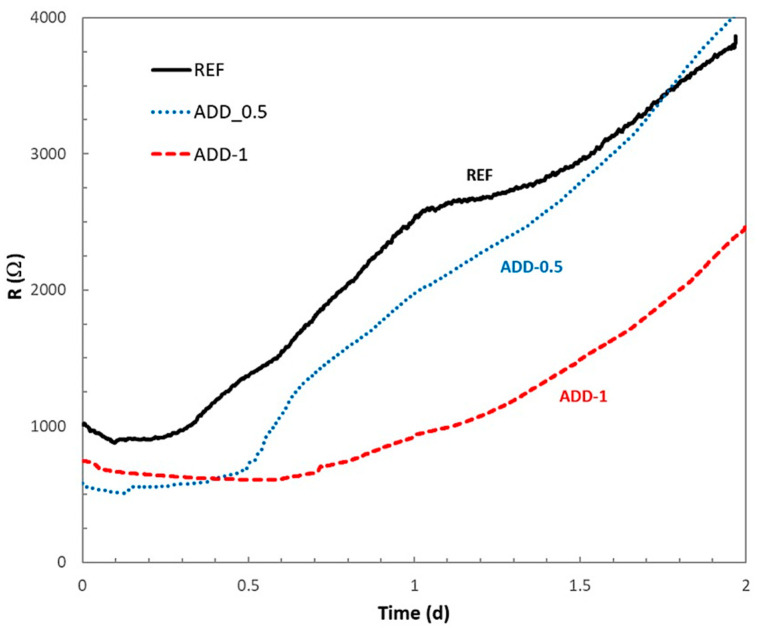
Evolution of the electrical resistance of the different mortars during the first 2 days curing in chamber at 98% relative humidity.

**Figure 8:**
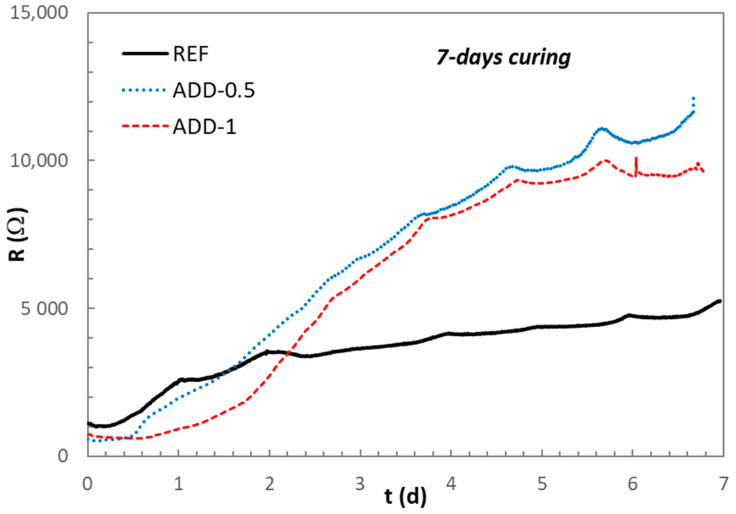
Evolution of the electrical resistance of the different mortars during the first 7 days curing in chamber at 98% relative humidity.

To evaluate whether the delay in the hydration processes is associated with the presence of the additive is maintained over time, the evolution of the electrical resistance was monitored up to 2 and 7 days of hardening under high relative humidity conditions. The mean value of the electrical resistance for three sensors embedded in the different mortars studied, monitored up to 2 and 7 days of curing, is shown in Figure 7 and Figure 8, respectively.

The electrical resistance values of the mortars after the first 2 days of hardening in high-humidity conditions, Figure 7, shows a continuous increase with time, maintaining higher values of resistance for the reference mortar without crystalline admixtures. In the case of a higher additive content (ADD-1) a slower increase is recorded, with electrical resistance values remaining below those of the reference mortar during the whole registered period. In the case of a lower content of crystalline admixture (ADD-0.5), the electrical resistance values show more similar values than those of the reference case after about 1.5 days of curing. It is to be expected that the crystalline additive in this case will not affect the hydration processes after this time. However, when a higher admixture content is added, the action of the admixture on the formation of the hydrated phases of the cement paste is still active.

After 7 days of hardening, the electrical resistance values of the mortars with additive reach higher values than those of the reference mortar, as can be seen from Figure 8, indicating that the effect of the additive in the hydration of the cementitious phases is not observed anymore. A more developed microstructure of the solid matrix with reduced pore size and lower connectivity can be expected after 7 days of hardening in the mortar incorporating the crystalline admixtures [24].

**Figure 9:**
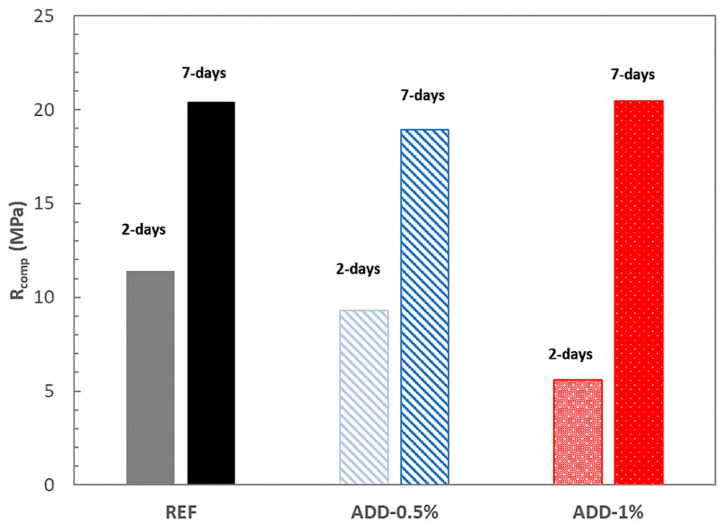
Compressive strength of the mortars cured in high-humidity conditions.

**Figure 10:**
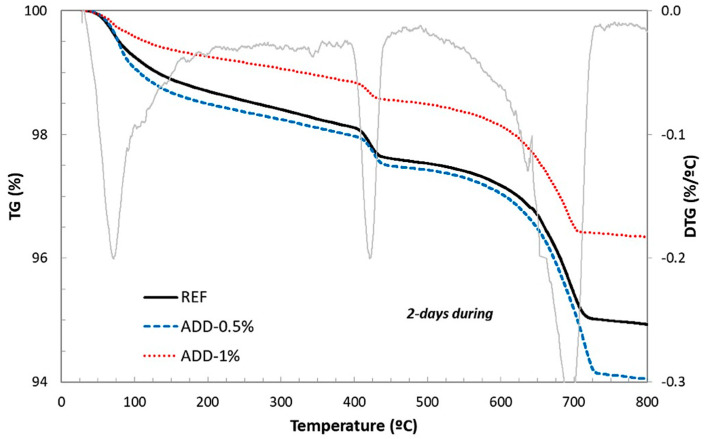
Thermogravimetric analyses of specimens of the studied mortars after 2 days curing in high-humidity conditions (98% relative humidity).

These results were confirmed both with the compression resistance measures and with the thermogravimetric analysis, as can be observed from Figure 9 and Figure 10, respectively.

The compression resistance of the mortar after 2 and 7 days of curing in high-humidity conditions is shown in Figure 9.

In the case of mortars with additives, a delay in the development of mechanical strength is registered when 1% of crystalline additive (ADD-1) is incorporated, probably justified by the retard in developing the hydrated phases of the cementitious matrix deduced from the monitoring of the electrical resistance values. However, no significant effect is observed on the mechanical properties of the mortar when a lower addition of crystalline admixture is incorporated to the mix, as can be observed from Figure 9.

An approximative estimation of the content of hydrated phases in the cementitious matrix of the studied mortars can be obtained from thermogravimetric analysis, as shown in Figure 10.

The effect of the presence of crystalline admixture in the matrix can be deduced from the lesser weight loss registered in the case of mortar ADD-1, indicating a slower development of hydration reactions. Almost no influence is observed in the case of mortar with ADD-0.5. The grey line in the graph shows the DGT curve for the reference mortar, indicating the temperatures at which the most significant weight losses occurs. The DGT curve obtained for the mortars with additives was similar, thus these curves are not shown in the figure for better visibility.

An indirect estimation of the hydrated phases of the cementitious matrix can be obtained from the TG data [36]. Three main regions can be identified in the TG curves, corresponding to: (a) between 100 and 300 °C attributed to the dehydration of C-S-H gel and ettringite, (b) a zone ranging between 390 and 480 °C, attributed to the dehydration portlandite, (c) and a region above 600 °C where the decomposition of carbonates occurs. The total loss of water coming for the hydrated phases can be obtained from the difference of weight between 100 and 600 °C [37].

**Figure 11:**
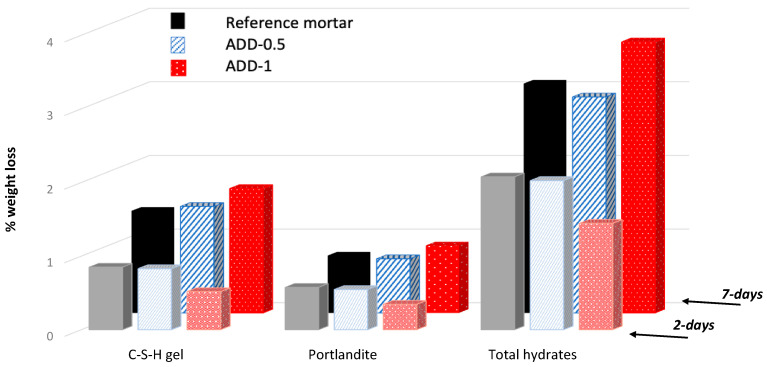
Estimation of the content of hydrated phases after 2 and 7 days of curing in high-humidity conditions (98% relative humidity).

As can be confirmed from Figure 11, the thermogravimetric results support the hypothesis of lesser hydration when 0.9% crystalline admixture (ADD-1) is incorporated to the mix that was also deduced from the electrical resistance results. A more similar hydration degree than in the case of reference mortar is estimated when 0.45% crystalline additive (ADD-0.5) is present in the mortar after 2 days of curing, as expected from the mechanical properties of the mortar. After 7 days of hardening the hydration is similar between the reference mortar and the mortar with 0.5% additive, and even higher in the case of 1% additive, confirming the results obtained by the electrical resistance monitoring and by the compression resistance measurements.

### 3.4. Waterproofing Performance of Crystalline Admixtures after 7-Days Curing

**Figure 12:**
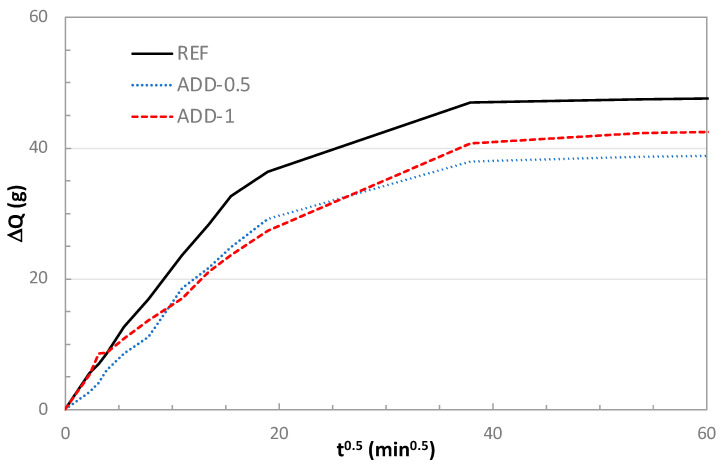
Water capillary absorption test in mortars after 7 days of hardening under high-humidity conditions (>98% RH).

The capillary water absorption of the waterproofed mortars incorporating crystalline admixtures was evaluated after 7 days of curing under high-humidity conditions (>98% RH). In Figure 12 the water uptake with the square root of time is represented for the three different mortars studied.

The time until saturation (t_n_) and the water capillary absorption (k) coefficient were estimated from the water capillary absorption test and are summarized in Table 3.

Mortar containing the crystalline admixture in the composition shows a higher resistance against the entrance of water by capillary absorption, with longer times until saturation. This effect is more significant for the higher content of crystalline admixture. The water capillary absorption coefficient is lower in the case of mortar with crystalline additives, with a more notable effect for the higher content of crystalline admixture.

## 4. Conclusions

The incorporation of crystalline admixtures in cement-based mortars delays the onset of setting and causes a delay in the development of the cement hydration processes. This effect, depending on the amount of admixture added, can maintain curing times even longer than 2 days.

Continuous monitoring of the electrical resistance from embedded sensors proved to be a useful tool to identify the effect of the crystalline admixture on the behavior of the fresh state and the first days of curing of the mortars.

After 7 days of mortar curing in low-humidity conditions, the material properties are no longer affected by the presence of the additive, showing even higher compressive strength and waterproofing efficiency when the crystalline additive is present in the mix.

The measurements allow to evaluate the influence of the amount of admixture used, thus being very useful as a tool to define the optimum admixture dosage to be used.

## Figures and Tables

**Table 1:** Mortar mix proportion of the studied samples (in g/mold).

Sample Name	Cement	Sand	Water	CA	w/c Ratio
REF	450	1350	225	---	0.5
ADD-0.5	450	1350	225	2	0.5
ADD-1	450	1350	225	4	0.5

**Table 2:** Electrical resistance values of sensors at different hydration times: initial and after 1 day.

Sample Name	R_initial_ (Ω)	R_1day_ (Ω)	Increment (Ω)
REF	1020 ± 534	2174 ± 486	1154 ± 48
ADD-0.5	568 ± 260	1973 ± 410	1405 ± 150
ADD-1	747 ± 243	929 ± 210	182 ± 33

**Table 3:** Parameters estimated from water capillary absorption test: saturation time and water capillary absorption coefficient.

Sample Name	t_n_ (min)	k (kg·m^−2^·min^−0.5^)
**REF**	556	0.0105
**ADD-0.5**	576	0.0082
**ADD-1**	817	0.0076

## Data Availability

Not applicable.
